# Virtual Healthcare Center for COVID-19 Patient Detection Based on Artificial Intelligence Approaches

**DOI:** 10.1155/2022/6786203

**Published:** 2022-01-11

**Authors:** Seifeddine Messaoud, Soulef Bouaafia, Amna Maraoui, Lazhar Khriji, Ahmed Chiheb Ammari, Mohsen Machhout

**Affiliations:** ^1^Laboratory of Electronics and Microelectronics, Faculty of Sciences, University of Monastir, Monastir, Tunisia; ^2^Department of Electrical and Computer Engineering, College of Engineering, Sultan Qaboos University, Muscat, Oman

## Abstract

At the end of 2019, the infectious coronavirus disease (COVID-19) was reported for the first time in Wuhan, and, since then, it has become a public health issue in China and even worldwide. This pandemic has devastating effects on societies and economies around the world, and poor countries and continents are likely to face particularly serious and long-lasting damage, which could lead to large epidemic outbreaks because of the lack of financial and health resources. The increasing number of COVID-19 tests gives more information about the epidemic spread, and this can help contain the spread to avoid more infection. As COVID-19 keeps spreading, medical products, especially those needed to perform blood tests, will become scarce as a result of the high demand and insufficient supply and logistical means. However, technological tests based on deep learning techniques and medical images could be useful in fighting this pandemic. In this perspective, we propose a COVID-19 disease diagnosis (CDD) tool that implements a deep learning technique to provide automatic symptoms checking and COVID-19 detection. Our CDD scheme implements two main steps. First, the patient's symptoms are checked, and the infection probability is predicted. Then, based on the infection probability, the patient's lungs will be diagnosed by an automatic analysis of X-ray or computerized tomography (CT) images, and the presence of the infection will be accordingly confirmed or not. The numerical results prove the efficiency of the proposed scheme by achieving an accuracy value over 90% compared with the other schemes.

## 1. Introduction

The new coronavirus disease (COVID-19) appeared at the end of December 2019 in Wuhan city of China and has affected most of countries around the world [1, 2]. COVID-19 is caused by the novel severe acute respiratory syndrome coronavirus 2 (SARS-CoV-2) and represents the causative agent of a potentially fatal disease, making it a global public health concern. In this context, person-to-person COVID-19 infection transmission has led to the isolation of patients who are subsequently administered a variety of treatments. In general, COVID-19 is an acutely resolved disease, but it can also be fatal, with a high fatality rate.

As of August 25, 2020, nearly 815,113 COVID-19 victims have died, while the total number of infected subjects was approximately 23,652,302 cases. This is caused by SARS-COV-2, currently spread all over the world [3]. The outbreak began in Mainland China, with a geographic concentration in Wuhan City, Hubei. However, on February 26, 2020, the rate of increase in cases became higher in the rest of the world compared to that in China. Large outbreaks are occurring on a daily basis in Italy (69,176 cases), the United States (51,914 cases), and Iran (24,811 cases), and the geographic spread of the epidemic continues. The respiratory transmission of the disease from one person to another has caused the epidemic to spread rapidly, where the most common infection signs include respiratory issues, fever, cough, symptoms, and dyspnea.

Given the high number of infections and blockages at the hospital level, this disease can be mortal as a result of delayed detection and progressive respiratory failure [3]. Therefore, automatic and early COVID-19 diagnosis using advanced artificial intelligence (AI) techniques can aid countries in terms of rapid patient quarantining and disease monitoring. Neither chemicals nor enormous medical capabilities are required to perform the tests, and data analysis does not require long periods.

In this vein, deep learning, an important breakthrough in the AI field, has shown enormous potential for extracting tiny features in image analysis [4]. Because of the COVID-19 pandemic, some deep learning approaches have been proposed to detect patients infected with the coronavirus. Loey et al. developed a deep learning system based on a generative adversarial network (GAN) with deep transfer learning for coronavirus detection in chest X-ray images [5]. To recognize COVID-19, the authors of [6] proposed a transfer-learning algorithm with convolutional neural networks using X-ray images. The authors of [7] proposed deep long short-term memory (LSTM) technique-based COVID-19 analysis that can recognize positive COVID-19 cases from both negative and healthy COVID-19 cases via cough and breath recordings on smartphones or wearable sensors. A deep learning technique-based early screening model was proposed by Xiaowei et al. [8] to detect COVID-19, Influenza, and viral pneumonia using computerized tomography (CT) images. The authors of [9] proposed fast algorithm-based deep learning to extract COVID-19 features to be used for diagnosis before pathogenic tests, which saves critical time. In addition, a deep learning-based model for detecting novel coronavirus pneumonia on high-resolution CT was suggested in [10]. Therefore, according to the development of intelligent techniques, future research directions may include more robust training strategies, for example, combining GAN with a genetic algorithm, incorporating edge and texture enhancement, reducing possible hallucination, coupling with explainable AI (XAI) modules, and exploring uncertainty measures in Bayesian deep attentive neural networks.

In summary, these deep learning tools-based models, which use either CT images or X-ray images, focus only on COVID-19 detection at the lung level, which is inconvenient for large populations as it requires scans of individual patients to detect the disease. In addition, the disease may be in the incubation period, which means it might be undetected by X-rays and limited to the lung levels. To address this problem, the main contribution of this paper is to propose an innovative healthcare center to provide a COVID-19 disease diagnosis (CDD) framework based on deep learning techniques. However, our CDD framework considers both CT and X-ray images and provides a screening phase for the patient's symptoms before proceeding to the detection phase. The proposed framework implements two modules. First, a patient's symptoms checker (PSC) based on the regression technique is developed to provide automatic initial medical screening for patients. This module predicts how much a patient is likely to be infected based on their symptoms. Conforming to the infection probability results obtained, X-ray or CT images are taken. These images are then used by the second module, which performs deep learning-based COVID-19 automatic detection to confirm or infirm the presence of the infection. An application is developed to experiment with both modules at the cloud level. In this paper, the proposed novel framework is explained in detail, and experimental validations are performed.

The remainder of this paper is organized as follows. In Section 2, the related work to COVID-19, deep learning techniques, and medical images will be provided. Then Section 3 discusses the architecture of the proposed deep learning-based CDD tool. In Section 4, we describe the collection of the COVID-19 dataset of the proposed framework.  Section 5 discusses the experimental results of the proposed scheme. Lastly, the conclusion of this paper is provided in Section 6.

## 2. Related Work

Unlike bacterial pneumonia, other types of lung infections are caused by viruses and are commonly called viral pneumonia. These viruses infect the lungs by blocking the oxygen flow, which can be life-threatening. COVID-19 is a recent example, defined as viral pneumonia caused by a new coronavirus that has quickly spread around the world and infected human life [11, 12]. In this context, SARS [13], COVID-19, and MERS [14] are studied as serious types of viral pneumonia which have spread continually to the pandemic level. Therefore, the development of an intelligent technique for viral pneumonia detection and infected cases recognition is recommended.

During the COVID-19 pandemic, viral nucleic acid detection using real-time polymerase chain reactions (RT-PCR) is the standard diagnostic method [15, 16]. Therefore, several hyperendemic nations are notable for providing sufficient RT-PCR tests for a million suspect cases in a short period. However, this method fails to recognize the new coronavirus before extracting the new virus's DNA sequence. Medical imaging is the most important method to help physicians assess disease development and take preventive measures as rapidly as possible. Clinically, chest radiography is the most widely used imaging modality in the patient's diagnosis of thoracic abnormalities given its effectiveness and significantly low cost [17]. Compared with a CT scan, an X-ray image cannot provide a 3D image of the human body's anatomy, but it is able to distinguish between viral pneumonia and nonviral pneumonia. In this context, a group of authors retrospectively studied the relationship between the chest CT results of examinations and clinical symptoms, laboratory tests, and other clinical factors for RT-PCR-positive cases. They used a machine learning (ML) method-based systematic decision support system to predict the chest CT results for RT-PCR-positive pediatric patients. Their proposed system achieved an AUC of 0.84 with 0.82 accuracy and 0.84 sensitivity for predicting CT outcomes. Similarly, another recent study was presented for the automatic segmentation of high-intensity scar tissue and left atrium anatomy in the 3D LGE-CMR images of atrial fibrillation patients. For the segmentation of both the scar tissue and the left atrium, a method based on a multiview two-task (MVTT) recursive attention model was proposed, which consists of three subnetworks incorporating multiview learning, ConvLSTM, and an attention mechanism. The mean dice scores of 93% and 87% were obtained for LA anatomy and scar tissue, respectively.

Typically, radiography is a technique used to quantify the functional and structural consequences of chest disease, with the aim to provide high-resolution images of the disease progression. Several works have been carried out in this context. Pneumonia incidence detected via X-ray imaging was documented by the authors of [18] to be guided by subsequent vaccine treatment. In [19], the contributors described the most common lung abnormality patterns in COVID-19 using chest X-rays and affirmed that the medical community can rely on chest X-rays because of their wide availability and reduced problems. The authors of [20] have proven that common CT findings of peripheral distribution and bilateral involvement can also be seen on chest X-rays, showing the potential of chest X-rays as a powerful tool for COVID-19 recognition. Another experimental chest X-ray scoring system was proposed in [21] to be applied to hospitalized, infected patients with COVID-19.

Unlike these studies, we aim to screen for viral pneumonia and to develop a rapid and intelligent scheme based on deep learning techniques to differentiate infected patients with viral pneumonia from uninfected patients for the control and prevention of a possible epidemic.

Recent advancements in AI (deep learning) have made it possible to break into many long-standing medical image analysis tasks, such as monitoring, detecting, recognizing, and delineating pathological abnormalities. In the task of interpreting X-rays, deep convolutional nerve networks (DCNNs) have been established to diagnose and identify the most common and important chest diseases [22–25] and to distinguish between viral pneumonia and bacterial pneumonia [26, 27]. In this context, many scientific researchers have used deep learning for the interpretation of radiographic images, with the aim of improving efficiency and reducing the burden on radiologists. The authors of [22] proposed a supervised framework for the classification and localization of fourteen common chest diseases. In [23], 121 dense convolutional neural network layers have been created to perform recognition tasks better than a radiologist. An awareness mechanism was proposed by the authors of [24] to help the model focus on the lesion area and thus further improve diagnosis performance. This is done using the correlation between class labels and pathological abnormality locations and by analyzing the feature maps learned by the classification branch. In addition, numerous attempts [26, 27] have been made to detect and differentiate between viral pneumonia and bacterial pneumonia based on DCNN classification models.

However, researchers have argued that many ML and deep learning-based models have major drawbacks because of flawed methodologies, underlying high biases in data collected from numerous public data repositories that have little verification opportunity, inadequate validation using external datasets that lack generalizability, and the insufficient documentation of publicly available datasets and codes for replicability. Thus, high-quality data, robust methodologies, demonstrations of strong validation using internal and external datasets and external models, and the availability and sufficient documentation of public datasets and codes are necessary to develop a trusted and dependable model that can provide consistent accuracy across seen and unseen data and contexts.

The main contribution of this paper is to propose an innovative healthcare center to provide a CDD framework based on deep learning techniques. However, the proposed CDD framework considers both CT and X-ray images and provides a screening phase for a patient's symptoms before proceeding to the detection phase.

## 3. Proposed Healthcare Center Framework Based on Deep Learning Models

**Figure 1:**
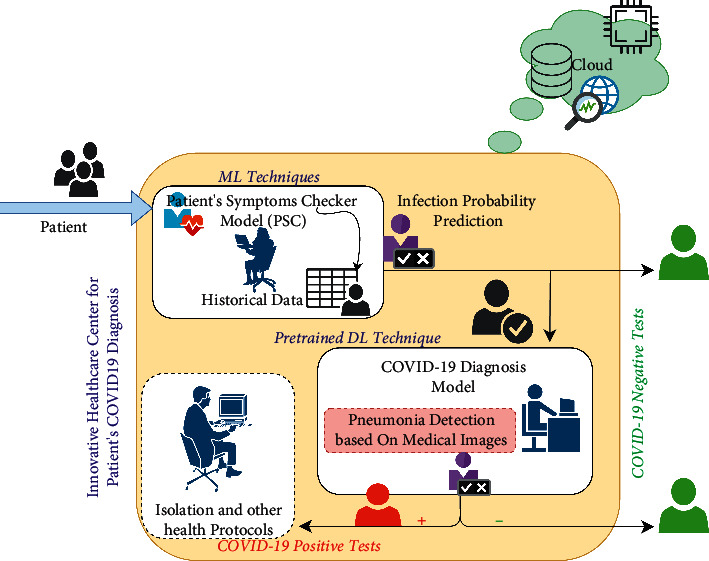
Proposed healthcare center for patient's COVID-19 diagnosis.

Early warning and prediction is one of the most powerful technologies to fight the COVID-19 pandemic. In this context, the proposed healthcare center for CDD implements two phases as well as PSC and COVID-19 diagnosis. However, at the first stage, patients will undergo the symptoms checking phase with the aim to predict how much they are likely to be infected based on these symptoms. Then, conforming to the obtained infection probability results, X-ray or CT images at the lung level are taken. These images are then used by the second module, which performs deep learning-based COVID-19 automatic detection to confirm or infirm the presence of the infection. ML and deep learning techniques [28] are used in this contribution. The following sections will provide a detailed overview of the models that will then be implemented in the healthcare center, as shown in Figure 1.

### 3.1. Patient's Symptoms Checker (PSC) Model Based on ML Techniques

**Figure 2:**
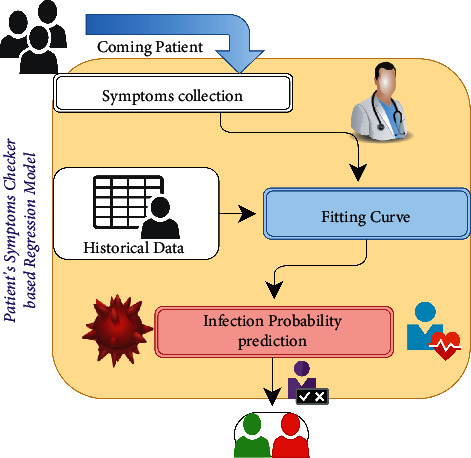
PSC based on regression model.

COVID-19 is mainly transmitted by droplets generated when an infected person coughs, sneezes, or exhales [29]. However, these droplets are too heavy to hang in the air and quickly fall to floors or surfaces. The infection can then be spread by breathing in the virus close to an infected patient or by touching a contaminated surface. In more severe cases, the infection can cause acute respiratory syndrome, septic shock, viral pneumonia, multiorgan failure, and death. In this context, we assume that patients will undergo symptoms checking for the first time to predict the possibility of COVID-19 infection, as illustrated in Figure 2. However, the proposed PSC module can predict infection probability based on the patient's symptoms and ML techniques.

Before introducing the main system based on the ML tools, we review some popular algorithms. Then, based on their performances, one of them will be selected to be the brain of the symptoms checker model. Among these commonly used algorithms in many domains, we choose the KNN, SVM, and logistic regression methods.

#### 3.1.1. KNN Technique

The K-nearest neighbors (KNN) technique is a supervised ML method that can be used for both classification and regression predictive problems [30]. However, this is mainly used for the classification of predictive problems in several areas. The two following properties would define KNN well:

- Lazy learning algorithm: KNN is a lazy learning algorithm because it does not have a specialized training phase and uses all the data for training during classification.
- Nonparametric learning algorithm: KNN is also a nonparametric learning algorithm because it does not assume anything about the underlying data.

The KNN algorithm uses feature similarity to predict the values of new data points, which further means that the new data point will be assigned a value based on how closely it matches the points in the training set.

#### 3.1.2. SVM Technique

Support vector machines (SVM) are powerful yet flexible supervised ML algorithms that are used for both classification and regression [31], but, generally, they are used in classification problems. SVM techniques have their unique ways of implementation as compared to other ML techniques. Lately, they have become extremely popular because of their ability to handle multiple continuous and categorical variables. An SVM model is basically a representation of different classes in a hyperplane in a multidimensional space. The hyperplane will be generated in an iterative manner via SVM so that the error can be minimized. The goal of SVM is to divide the datasets into classes to find a maximum marginal hyperplane (MMH).

#### 3.1.3. Logistic Regression Technique

Logistic regression is a supervised learning classification algorithm used to predict the probability of a target variable [32, 33]. The nature of the target or dependent variable is dichotomous, which means there would be only two possible classes. Hence, the symptoms are considered as the input for the SPC model. In this context, let us denote by *X*_*m*_ = {*x*_*i*_,…, *x*_*n*_} the symptoms vector, and let *y*_*m*_ be the predicted infection probability, where *m* ∈  {1,…, *M*} represents the data length (*M* patients, each having *i* symptoms). The relationship between *X* and *y* is then modeled by the following equation, where *β* represents the regression coefficient.  Algorithm 1, summarizes the ML technique implementation.

(1) $$\begin{array}{c} y_{i}=\frac{1}{1+\exp\left(-\left(\beta_{0}+\beta_{i}x_{i}+\cdots +\beta_{n}x_{n}\right)\right)}. \end{array}$$

### 3.2. COVID-19 Diagnosis Based on DL Model

In this section, the VGG19 model used for COVID-19 detections will be described first. Then the proposed COVID-19 diagnostic scheme based on the pretrained CNN model will be introduced. Subsequently, before proceeding to the learning phase and the experimental results, the used datasets in this simulation will be clarified.

#### 3.2.1. Deep VGG Model

In this study, we explain in detail the selected pretrained VGG19 model and its structure. VGGNet was invented by Simonyan and Zisserman [34] at the University of Oxford Robotics. This deep network architecture has two versions with different depths and layers, namely, VGG16 and VGG19. In this work, VGG19 was used to achieve the clinical purpose of the COVID-19 model.  Table 1 summarizes the VGG19 layers and parameters. The VGG19 architecture consists of five blocks of convolutional layers; each block is followed by a pooling layer and three fully connected layers at the end. At first, the input RGB image to the network is of the size of 224 × 224 × 3, which passes through five convolutional layers with a kernel size of 3 × 3 and a stride of one. After the convolutional layers, the maxpooling layers are performed over a 2 × 2 kernel size with a stride of two. In the end, the last pooling layer is followed by the flattening layer and three fully connected layers for COVID-19 detection.

#### 3.2.2. COVID-19 Detection Based on VGG19 Model

**Figure 3:**
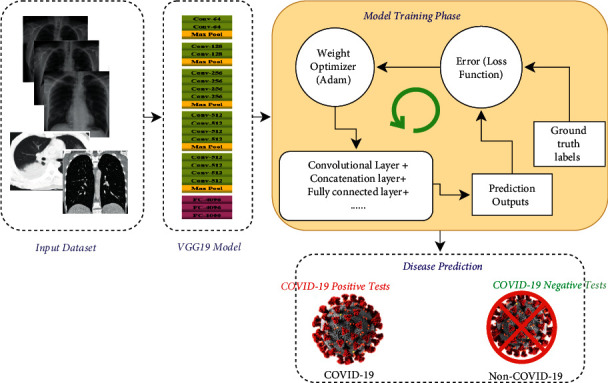
VGGNet model for COVID-19 diagnosis.

To detect COVID-19 from non-COVID-19 cases, we proposed a deep learning method in this study.  Figure 3 describes the proposed method based on the deep CNN model; in particular, we have chosen VGG19. As shown in Figure 3, we developed a deep CNN-based VGG19 model for COVID-19 detection tasks (COVID-19 and non-COVID-19 classes) using medical images. In the first stage, the medical images collected in the dataset can be processed later in the deep learning pipeline, loaded for a fixed size of 224 × 224. Then, to denote the positive case (COVID-19) or “not” for each subject in the dataset, we applied one-hot encoding on the labels of image data. In the second stage (the training phase), the preprocessed dataset will be divided into 80% for constructing equal training and validation sets and 20% for the testing phase. The selection of training data for the deep classifier will be a random subsample. Hence, to measure the performance of the validation set, we apply the evaluation metrics illustrated in the next section. In the final stage, the categorization of all images, classified into two categories—positive COVID-19 or non-COVID-19—is performed by transmitting the testing data.

## 4. Dataset Collection

### 4.1. Symptoms Dataset

To prevent the spread of the coronavirus and possibly help treat the disease before it gets worse, we have proposed an early detection method for COVID-19 by checking symptoms. In fact, a wide range of symptoms, ranging from mild to serious, appear within two to fourteen days of the virus infecting people with COVID-19. Among these symptoms, we can cite the following: shortness of breath or difficulty breathing, repeated shaking with chills, cough, fever, muscle pain, chills, sore throat, new loss of taste or smell, and headache [35].

In this context, for the automatic symptoms checker module, we used a public day-level information dataset on COVID-19 cases [35]. The dataset used in this model to train ML techniques consisted of 270 patients, each with their ID, gender, and age and the discussed symptoms. Each subject in the dataset, based on their symptoms, is classified as 0 to denote a normal case or 1 to denote an infected case.  Table 2 further clarifies the dataset.

### 4.2. COVID-19 Dataset

The proposed deep learning model was trained and tested based on two public, open datasets of X-ray and CT images. Therefore, the first dataset consists of Computed Tomography (CT) images of normal patients and patients infected with COVID-19, which are taken from the GitHub repository [36]. From [37, 38], we selected an X-ray dataset containing normal patients and COVID-19 patients.

In many countries, hospitals do not include both CT and X-ray machines. In this case, the proposed CDD framework can support medical images diversity and can deal with both CT image and X-ray images. On the other hand, DNN requires a large database in the training phase to achieve a high testing accuracy level and solid model that can correctly do the prediction. However, we have combined these two datasets as shown in Table 3.

The proposed deep learning model was trained and tested based on two public open datasets of X-ray and CT images. Therefore, the first dataset consists of the CT images of normal patients and patients infected with COVID-19, which are taken from the GitHub repository [36]. From [37, 38], we selected an X-ray dataset containing normal patients and COVID-19 patients.

In many countries, hospitals do not have both CT and X-ray machines. In this case, the proposed CDD framework can support medical image diversity and can deal with both CT and X-ray images. On the other hand, DNN requires a large database in the training phase to achieve a high testing accuracy level and a solid model that can correctly do the prediction. As such, we have combined these two datasets, as shown in Table 3.

**Figure 4:**
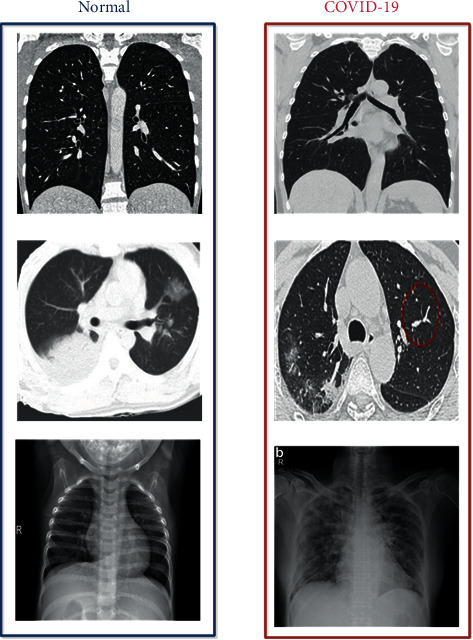
Example of X-ray and CT images of a normal patient and a COVID-19 patient.

Our main goal here is to achieve good results in detecting COVID-19 cases. In this work, the datasets are gathered into two subsets, 80% for training and 20% for testing, including normal and COVID-19 cases.  Figure 4 shows the CT and X-ray images.

### 4.3. Evaluation Metrics

Regarding the logistic regression (VGG19) prediction (classification) task, specific metrics are denoted as follows: incorrectly predicted (classified) healthy cases (False Negatives, FN), incorrectly predicted (classified) diseased cases (False Positives, FP), correctly identified healthy cases (True Negatives, TN), and correctly identified diseased cases (True Positives, TP). Based on these metrics, we use equations (1) to (4) to calculate the accuracy, precision, recall, and F1-score, respectively.

(2) $$\begin{array}{c} \text{Accuracy}=\frac{\text{TP}+\text{TN}}{\text{TP}+\text{FP}+\text{FN}+\text{TN}}, \end{array}$$

(3) $$\begin{array}{c} \text{Precision}=\frac{\text{TP}}{\text{TP}+\text{FP}}, \end{array}$$

(4) $$\begin{array}{c} \text{Recall}=\frac{\text{TP}}{\text{TP}+\text{FN}}, \end{array}$$

(5) $$\begin{array}{c} \text{F}1\_\text{score}=2\times \frac{\text{Recall}\times \text{Precision}}{\text{Recall}+\text{Precision}}. \end{array}$$

## 5. Numerical Results

The experimental results have been introduced in this section to evaluate our suggested framework based on ML/DL techniques. We begin by evaluating the symptoms checker model by selecting the most effective ML technique. Thereafter, we evaluate the performance of the proposed COVID-19 diagnosis model by exploiting CT images, then using X-ray images, and finally by using both in the same dataset.

### 5.1. Regression Technique-Based Symptoms Checker Model

To select one of the suggested algorithms as the brain for our symptoms checker model, we implement different ML techniques (KNN, SVM, and logistic regression) and then evaluate their performance metrics. Here, we consider the explained dataset, consisting of the patient's ID and symptoms input, where the output is labeled “0” to indicate that the patient is not infected (low infection probability *≤*40%) and “1” to indicate that the patient is infected (high infection probability *≥*50%).  Table 4 further clarifies the dataset.

Table 4 shows that the logistic regression model used to check the subject's symptoms outperforms the other ML algorithms regarding their best performance in terms of accuracy, precision, recall, and F1-score. Overall, the logistic regression model achieves an average accuracy classification of 51.18% in two classes (COVID-19 and normal) compared with SVM and KNN, which achieved accuracy scores of 0.481 and 0.444, respectively. Regarding the other evaluation metrics, the logistic regression model reached the highest mean values for all classes. Based on these results, we consider the logistic regression technique as the brain tool to predict infection probability for patients.

**Figure 5:**
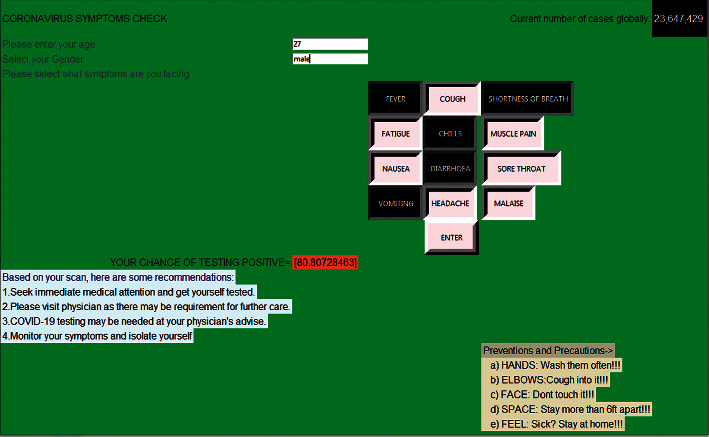
PSC application.

To assess the framework's symptoms checker, we created a test application that provides a small graphical interface for selecting symptoms and obtaining the predicted infection probability.  Figure 5 represents the graphical interface, created using Python, which contains symptom buttons; the doctor needs only to select the symptoms, and then the application will predict the infection probability of this patient (a positive case in the figure example). This interface also provides the total number of cases in the world from the website of the World Health Organization (WHO). Some advice will be provided when the symptoms test shows positive results. Note that this application also saves the patients' test results to be used as historical data to predict other cases and to increase the prediction accuracy.

### 5.2. VGGNet Technique-Based COVID-19 Diagnosis Model

The proposed deep learning model was trained and tested using the Python programming language [39]. The Adam optimizer technique is a powerful technique that updates the weights at each iteration and minimizes the gradient error between the ground truth labels and the prediction outputs [40]. In addition, the overall experiments were implemented on an Intel Core TM i7-3770 @3.4 GHz CPU and 16 GB RAM. We also use the NVIDIA GeForce RTX 2070 GPU to improve the speed of the proposed trained deep model. The deep learning parameters applied in this work are as follows. The number of epochs, the learning rate, and the batch size were experimentally set at 100, 0.0001 and 64, respectively. The datasets are gathered into two subsets, 80% for training and 20% for testing, including normal and COVID-19 cases.

During the training process, as shown in Figure 3, the cross-entropy is the loss function (*L*), mentioned in equation (6), used in this study, where *g* represents the ground truth labels and *p* denotes the prediction outputs.

(6) $$\begin{array}{c} L\left(g,p\right)=\frac{1}{M}\sum_{i=0}^{M}\left(g*\;\log\left(p_{i}\right)+\left(1-g\right)*\;\log\left(1-p_{i}\right)\right). \end{array}$$

The training performances in terms of the loss function, validation loss, accuracy rate, precision, recall, specificity, F1-scores, and validation accuracy for the deep learning model will be introduced for the X-ray dataset, followed by the CT dataset, and finally for the combined datasets.

#### 5.2.1. VGG19 Based on X-Ray Images

In this study, the performances of the proposed pretrained VGG19 model were evaluated using X-ray datasets to detect COVID-19.  Table 5 summarizes the performance evaluation of the proposed deep model in terms of precision, recall, specificity, and F1-score for the two classes. Based on the experimental findings, the proposed VGG19 classifier achieves a precision of 97% for the two classes, and the achieved recall values for the COVID-19 and normal classes are 96% and 98%, respectively. Further, the specificity reaches 98% for normal and COVID-19 cases, while the F1-scores for the COVID-19 and normal classes are 97% and 98%, respectively.

**Figure 6:**
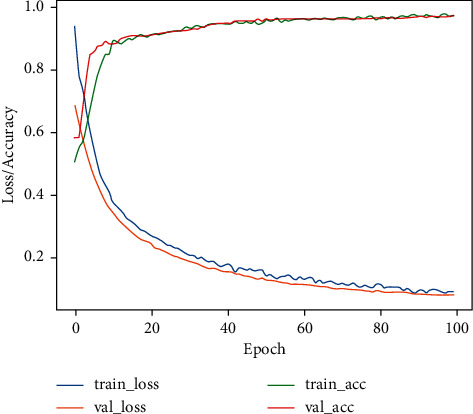
Accuracy and loss for COVID-19 detection.

**Figure 7:**
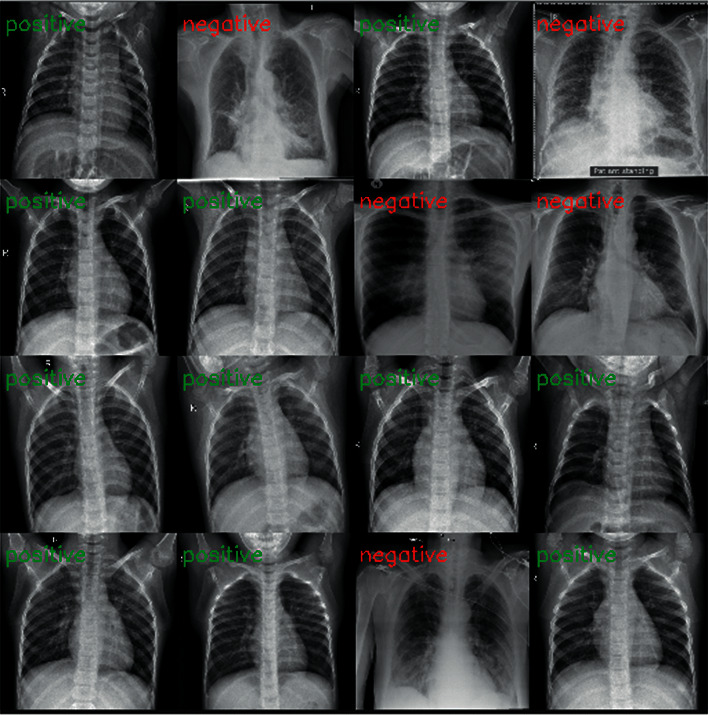
Tested VGG19 model.

Figure 6 shows the accuracy and loss curves on the training and validation processes to evaluate the performance of the proposed VGG19 network. The achieved training and validation accuracy rates are 97.3% and 97.1%, respectively. Similarly, the training and validation loss values were found to be 0.08 and 0.07, respectively, for the proposed model. Consequently, the proposed deep learning model reaches a good performance level of detection. For the testing phase, we tested our trained model to evaluate the VGG19 classifier for the detection of COVID-19 and normal cases on the X-ray datasets, as illustrated in Figure 7.

#### 5.2.2. VGG19 Based on CT Images

In this study, the performances of the proposed pretrained VGG19 model were evaluated using CT datasets to detect COVID-19.  Table 6 summarizes the performance evaluation of the proposed deep model in terms of precision, recall, specificity, and F1-score for the two classes. Based on the experimental findings, the proposed VGG19 classifier achieves an accuracy of 86% for normal and COVID-19 cases. The precision is 73% and 85% for the two classes, and the achieved recall values for the COVID-19 and normal classes are 86% and 72%, respectively. The specificity is 72% for both normal and COVID-19 cases, and the F1-scores for the COVID-19 and normal classes are 79% and 78%, respectively.

**Figure 8:**
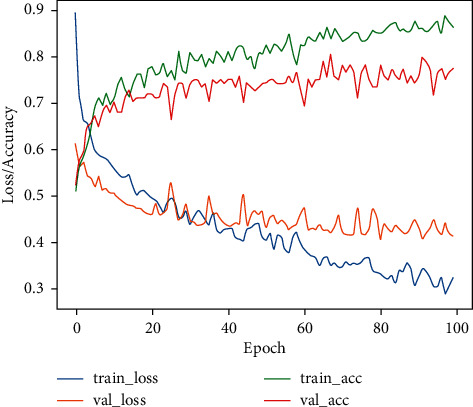
Accuracy and loss for COVID-19 detection.

**Figure 9:**
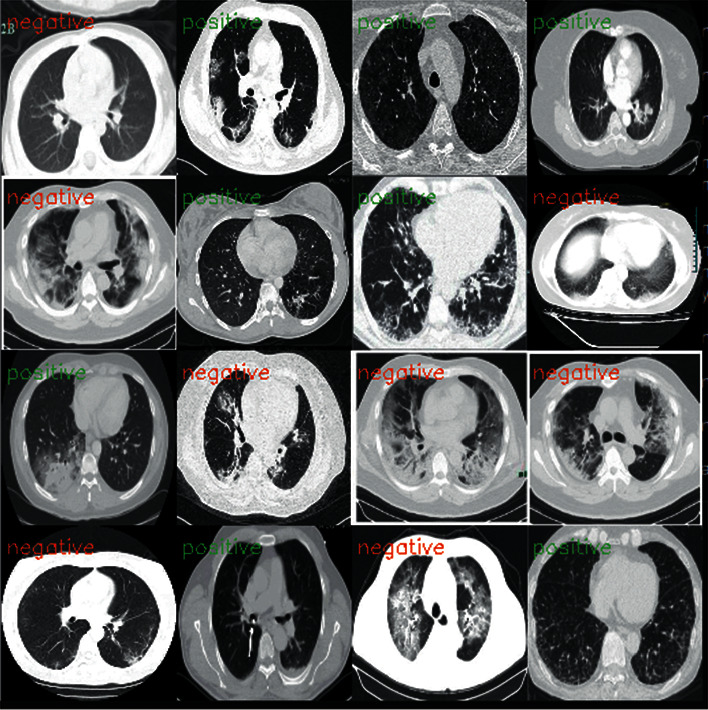
Tested VGG19 model.

Figure 8 shows the accuracy and loss curves on the training and validation processes to evaluate the performance of the proposed VGG19 network. The achieved training and validation accuracy rates are 86% and 77%, respectively. Similarly, the training and validation loss values are 0.32 and 0.41, respectively, for the proposed model. For the testing phase, we tested our trained model to evaluate the VGG19 classifier for the detection of COVID-19 and normal cases on the CT dataset, as illustrated in Figure 9.

#### 5.2.3. VGG19 Based on Combined Datasets

In this study, the performances of the proposed pretrained VGG19 model were evaluated using the CT and X-ray datasets, including two classes (normal and COVID-19), to detect COVID-19.  Table 7 summarizes the performance evaluation of the proposed deep model in terms of precision, recall, specificity, and F1-score for the two classes. Based on the experimental findings, the proposed VGG19 classifier achieves a precision of 86% and 91% for the two classes, and the achieved recall value for both COVID-19 and normal class is 89%. The specificity is 89% for both normal and COVID-19 cases, and the F1-scores for the COVID-19 and normal classes are 87% and 90%, respectively.

**Figure 10:**
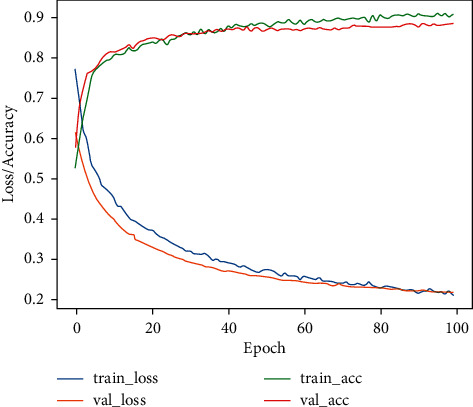
Accuracy and loss for COVID-19 detection.

**Figure 11:**
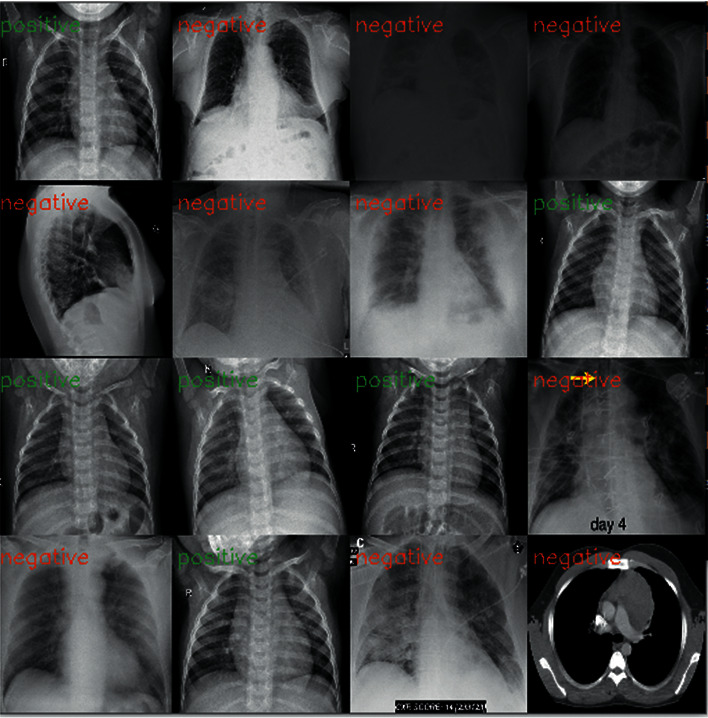
Tested VGG19 model.

Figure 10 shows the accuracy and loss curves on the training and validation processes to evaluate the performance of the proposed VGG19 network. The achieved training and validation accuracy rates are 90% and 88%, respectively. Similarly, the training and validation loss values are 0.20 and 0.21, respectively, for the proposed model. Consequently, the proposed deep learning model reaches a good performance level of detection. For the testing phase, we tested our trained model to evaluate the VGG19 classifier for the detection of COVID-19 and normal cases on the CT and X-ray datasets, as illustrated in Figure 11.

### 5.3. Comparative Study

For further evaluation, we have presented the performance comparison of the proposed VGG19 model with the advanced approaches, as shown in Table 8. From this table, we can conclude that the results obtained by our proposed deep learning model for COVID-19 detection are slightly superior to those of existing works in terms of evaluated performances. Regarding the accuracy value, our proposed model achieved the best accuracy rates of 97%, 86%, and 90% for normal and COVID-19 classes when using all three datasets—X-ray, CT, and both X-ray and CT, respectively. The authors of [41] developed a deep learning model—namely, COVID-Net—to detect COVID-19 and normal cases from X-ray images, and this model achieved an accuracy of 83.5%. Meanwhile, the authors of [42] proposed a CoroNet network for the detection of COVID-19 cases from X-ray images with an accuracy value of around 89.6%. Likewise, a VGG19 and DenseNet201 deep learning system achieved an accuracy value of 90% using X-ray datasets to classify COVID-19 and normal patients [43]. Moreover, to detect the two cases, the authors of [44] proposed a CNN architecture based on CT images which achieved 83% accuracy. We also compared our experiments with some relevant ML models, such as random forest (RF) (accuracy of about 95%), gradient boosting machine (GBM) (accuracy of about 92%), and KNN (accuracy of about 93%), proposed by [45]. In addition, the proposed technique was compared to the EfficientNet-B4 architecture, which uses a three-layer artificial neural network with an accuracy of about 83.43%, probably as a result of using fewer layers compared to our proposed scheme.

In summary, all the results of the experiments reveal that the proposed technique obtains good results in detecting COVID-19 cases compared with other related approaches. This comparative analysis and comparing the overall performance results achieved by the proposed deep CNN model provide strong evidence of the applicability of the model in COVID-19 diagnosis using X-ray and CT image classification.

## 6. Conclusion

SARS-CoV-2 is part of a large family of viruses that cause illnesses ranging from mild colds to fatal diseases. Thanks to advanced AI technology, the early detection of this virus will aid in the rapid recovery of COVID-19 patients. This paper proposed an innovative healthcare center that will be implemented in the cloud and gives access to doctors to diagnose and detect COVID-19 infection. We also propose a PSC based on a regression model to provide initial medical screening for patients and predict whether the patient is infected based on their symptoms. Therefore, based on the pretrained deep learning classifier VGG19, we propose a learning model to classify normal and COVID-19 cases using a dataset of X-ray and CT images. The performance results prove that the proposed scheme yields the highest accuracy of 90% compared with the others. We seek, in the near future, to add more classification classes such as bacterial pneumonia and other types of viral pneumonia.

## Figures and Tables

**Algorithm 1:**
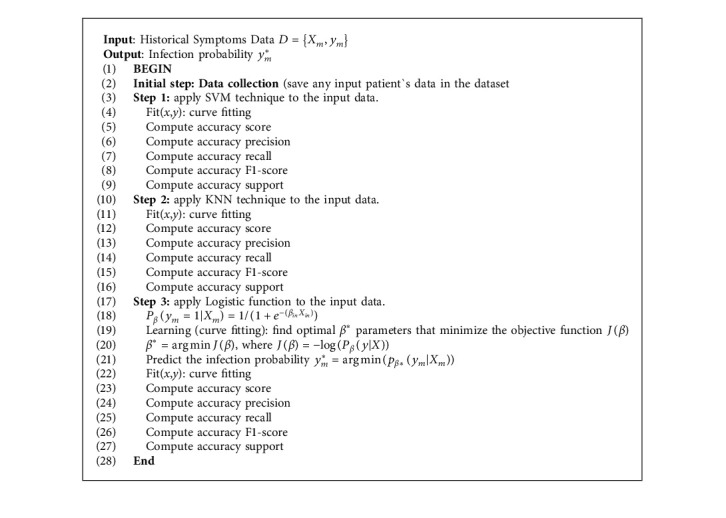
ML techniques for patient's symptoms checker.

**Table 1:** VGG19 model for the proposed scheme.

Layer (type)	Output shape	Parameters number
Input_1 (inputLayer)	(None, 224, 224, 3)	0
Block1_conv1 (conv2D)	(None, 224, 224, 64)	1792
Block1_conv2 (conv2D)	(None, 224, 224, 64)	36928
Block1_pool (maxPooling2D)	(None, 112, 112, 64)	0
Block2_conv1 (conv2D)	(None, 112, 112, 128)	73856
Block2_conv2 (conv2D)	(None, 112, 112, 128)	147584
Block2_pool (maxPooling2D)	(None, 56, 56, 128)	0
Block3_conv1 (conv2D)	(None, 56, 56, 256)	295168
Block3_conv2 (conv2D)	(None, 56, 56, 256)	590080
Block3_conv3 (conv2D)	(None, 56, 56, 256)	590080
Block3_conv4 (conv2D)	(None, 56, 56, 256)	590080
Block3_pool (maxPooling2D)	(None, 28, 28, 256)	0
Block4_conv1 (conv2D)	(None, 28, 28, 512)	1180160
Block4_conv2 (conv2D)	(None, 28, 28, 512)	2359808
Block4_conv3 (conv2D)	(None, 28, 28, 512)	2359808
Block4_conv4 (conv2D)	(None, 28, 28, 512)	2359808
Block4_pool (maxPooling2D)	(None, 14, 14, 512)	0
Block5_conv1 (conv2D)	(None, 14, 14, 512)	2359808
Block5_conv2 (conv2D)	(None, 14, 14, 512)	2359808
Block5_conv3 (conv2D)	(None, 14, 14, 512)	2359808
Block5_conv4 (conv2D)	(None, 14, 14, 512)	2359808
Block5_pool (maxPooling2D)	(None, 7, 7, 512)	0
Flatten (flatten)	(None, 25088)	0
Fc1 (dense)	(None, 4096)	102764544
Dropout (dropout)	(None, 4096)	0
Fc2 (dense)	(None, 4096)	16781312
Dropout_1 (dropout)	(None, 4096)	0
Fc3 (dense)	(None, 8192)	33562624
Dropout_2 (dropout)	(None, 8192)	0
Fc4 (dense)	(None, 8192)	67117056
Dropout_3 (dropout)	(None, 8192)	0
Fc5 (dense)	(None, 8192)	67117056
Dropout_4 (dropout)	(None, 8192)	0
Fc6 (dense)	(None, 16384)	134234112
Dropout_5 (dropout)	(None, 16384)	0
Dense_class_2 (dense)	(None, 2)	32770

**Table 2:** Symptoms dataset.

Patient ID	1	2	3	270
Male	0	0	1	0
Female	1	1	0	1
Age	28	51	37	25
Fever	1	1	1	0
Cough	1	1	0	0
Shortness of breath	1	1	0	1
Sore throat	0	0	0	0
Chills	0	0	0	0
Muscle pain	0	0	0	0
Nausea	0	1	0	0
Diarrhea	0	1	0	0
Fatigue	1	0	0	0
Vomiting	0	0	0	0
Headache	0	0	0	0
Malaise	1	0	1	1
Output (classes)	1	1	0	0

**Table 3:** X-ray and CT datasets.

Datasets	COVID-19	Normal
CT images	349	397
X-ray images	910	1341
Combined images	1259	1738

**Table 4:** Performance evaluation for machine learning models.

ML model	Accuracy score	Class	Precision	Recall	F1-score	Support
Logistic	0.518	0	0.86	0.33	0.48	18
regression		1	0.40	0.89	0.55	9

KNN	0.444	0	0.67	0.33	0.44	18
		1	0.33	0.67	0.44	9

SVM	0.481	0	0.75	0.33	0.46	18
		1	0.37	0.78	0.50	9

**Table 5:** Performance of VGG19 network based on X-ray dataset.

Metrics	COVID-19	Normal
Precision	0.97	0.97
Recall	0.96	0.98
Specificity	0.98	0.98
F1-score	0.97	0.98

**Table 6:** Performance of VGG19 network based on CT dataset.

Metrics	COVID-19	Normal
Precision	0.73	0.85
Recall	0.86	0.72
Specificity	0.72	0.72
F1-score	0.79	0.78

**Table 7:** Performance of VGG19 network based on combined dataset.

Metrics	COVID-19	Normal
Precision	0.86	0.91
Recall	0.89	0.89
Specificity	0.89	0.89
F1-score	0.87	0.90

**Table 8:** Performance comparison of the proposed VGG19 model with the state-of-the-art approaches.

Approaches	Model	Datasets	Class	Precision	Recall	F1-score	Accuracy
[41]	COVID-Net	X-ray	COVID-19	80	100	88.8	83.5
			Normal	95.1	73.9	83.17	

[42]	CoroNet	X-ray	COVID-19	93.17	98.25	95.61	89.6
			Normal	95.25	93.5	94.3	

[43]	VGG19+	X-ray	COVID-19	83	100	91	90
	DenseNet201		Normal	100	80	89	

[44]	CNN	CT	COVID-19	81.73	85	83.33	83
			Normal				

[45]	RF	X-ray	—	96	—	95	95
	GBM	X-ray	—	93	—	92	92
	KNN	X-ray	—	99	—	93	93

[46]	EfficientNet-B4+CLAHE	CT	—	86.81	78.27	82.32	83.43

Proposed	VGG19	CT	COVID-19	73	86	79	86
			Normal	85	72	78	
		X-ray	COVID-19	97	96	97	97
			Normal	97	98	98	
		CT + X-ray	COVID-19	86	89	87	90
			Normal	91	89	90	

## Data Availability

The data used to support the findings of this study are available from the corresponding author upon request.
